# Dietary diversity cutoff values predicting anemia varied between mid and term of pregnancy: a prospective cohort study

**DOI:** 10.1186/s41043-019-0196-y

**Published:** 2019-12-13

**Authors:** Taddese Alemu Zerfu, Kaleab Baye, Mieke Faber

**Affiliations:** 1 Center for Food Science and Nutrition, College of Natural Sciences, Addis Ababa University, Addis Ababa, Ethiopia; 2 Friedman School of Nutrition Science and Policy, Tufts University, Boston, USA; 3 College of Medicine and Health Sciences and Referral Hospital, Dilla University, Dila, Ethiopia; 4 Non-Communicable Diseases Research Unit, South African Medical Research Council, Cape Town, South Africa; 5 Maternal and Child Wellbeing Unit, African Population and Health Research Center (APHRC), P.O. Box 10787-00100, Nairobi, Kenya

**Keywords:** Dietary diversity, Cutoff, Metrics, Pregnancy

## Abstract

****Background**:**

Correcting anemia during pregnancy often requires integrating food and non-food-based approaches. Nonetheless, little is known about specific dietary diversity (DD) cutoff values predicting risk of anemia during the different trimesters of pregnancy.

****Objective**:**

We aimed to determine the lowest possible DD cutoff values associated with risk of maternal anemia at mid and term of pregnancy in a rural resource limited setting of Ethiopia.

**Design:**

A multi-center prospective cohort study was conducted enrolling 432 eligible pregnant women from eight rural health centers selected from four districts in Arsi zone, Central Ethiopia. Women were classified into exposed (*n* = 216) and unexposed (*n* = 216) groups, based on Women’s Individual Dietary Diversity (WIDD) score, and were followed from mid to term of pregnancy. The cutoff values for WIDD corresponding to the lowest risk of anemia were defined by receiver operating characteristic (ROC) curve analysis. Logistic regressions were also fitted to identify food groups associated with low anemia risk during pregnancy.

**Results:**

The overall prevalence of anemia increased from 28.6 to 32.4% between mid and term of pregnancy. Calculatedly, using the ROC curve analysis, the minimum WIDD score associated with lower risk of anemia was three and four respectively at these periods. Not consuming animal source foods [adjusted odds ratio (AOR), 2.36; 95% confidence interval (CI), 1.35–4.14], pre-existing anemia (AOR 28.56; 95% CI, 14.33, 56.79), and low DD during pregnancy (AOR, 2.22; 95% CI, 1.09–4.52) were associated with risk of anemia at term.

****Conclusion**:**

The cutoff for WIDD score predicting risk of anemia varied significantly, increasing from three to four, between mid and term of pregnancy. Additional population-based observational and experimental studies validating the metrics are needed before policy level recommendations.

**Trial registration:**

This trial was registered at clinicaltrials.gov as NCT02620943.

## Introduction

The first 1000 days of life, extending from conception to a child’s second birthday, is a unique period of opportunity when the foundations of optimum health, growth, and neurodevelopment across the lifespan are established [1, 2]. During this period, the essential building blocks for brain development, healthy growth, and a strong immune system are founded for continued development throughout life [3]. A growing body of scientific evidence also shows that some of the foundations of a person’s lifelong health risks and predisposition to chronic diseases are largely set during this period [4, 5].

On the other hand, regardless of maternal nutritional status during pregnancy, availability of essential nutrients to the fetus is maintained by the maternal hormonal system to a certain critical level [6, 7]. After the optimum regulation limit of these essential nutrients, however, the effectiveness of hormonal mechanisms decreases significantly, and the fetus will be predisposed for various adverse perinatal outcomes including lower birth weight, neurological disorders, impaired physical growth, mental retardation, and poor school performance [6]. The effect of marginal nutritional status during pregnancy could also potentially lower the quantity and quality of maternal milk production capacity [8].

Anemia, particularly iron-deficiency anemia (IDA) is the most common micronutrient disorder, negatively affecting the health and socio-economic wellbeing of billions worldwide [9–11]. Anemia during pregnancy increases risks of post-partum hemorrhage, low birth weight, small-for-gestational age, and perinatal death (still birth) [12, 13]. It also reduces fetal iron stores perhaps well into the first year of life [14, 15]. IDA alone contributes to over 100,000 maternal and 600,000 perinatal deaths each year globally [11, 15].

Although iron deficiency is the primary cause, IDA seldom presents in isolation, as other conditions such as malaria, parasitic infection, and deficiency of other nutrients strongly correlate or confound its presence [9, 16]. As such, correcting anemia often requires an integrated approach of combining both food-based and non-food-based interventions such as treatment of the underlying cause, restoration of the hemoglobin concentration to normal levels, and prevention and treatment of complications [10, 17, 18]. Nevertheless, in many of the Low- and Middle-Income Countries (LMIC), the control and prevention activities are largely dependent on non-food-based approaches like micronutrient supplementation and behavioral change interventions with little emphasis to dietary-related interventions [19–21].

A growing level of evidence indicates, lack of simple and low-cost metrics and methodological limitations in the state of knowledge about linkages between agriculture food systems, health, and nutrition outcomes have significantly affected the growing interest of donors, national governments, civil society, and private sector entities around the world to intervene on nutritional improvements [22, 23]. Dietary diversity scores (DDS) can be used as a simple measurement of dietary variety and have been shown to be associated with micronutrient adequacy of the diet [24, 25]. In the present analysis, we aimed to determine minimum DDS of women or formal called Women’s Individual Dietary Diversity (WIDD) cutoff values associated with lower risk of anemia at mid and term of pregnancy in resource limited settings of rural Ethiopia.

## Methods

### Study setting

Details on design, study area, and methodology of the study are reported elsewhere [26]. Here, we introduce briefly the study settings, sampling procedures, and data collection techniques employed for the present analysis. The study was conducted in eight randomly selected health centers of four rural districts that represent different agro-ecological zones of Arsi Zone, Oromia region, Central Ethiopia. The zonal Capital, Asella town, is located 165 km to the southwest of Addis Ababa. Arsi Zone is one of the surplus producing agricultural areas of Ethiopia with major production of wheat and barley. All forms of malnutrition including anemia are highly prevalent in the area, attributable mainly to lack of knowledge, dietary habits, food taboos, and misperceptions [27].

### Study design, sample size, and sampling procedure

A longitudinal prospective cohort study design was conducted among pregnant women enrolled during their first antenatal care visits that usually happened during the second trimester (median of 24 weeks) of gestation. Pregnant women in Ethiopia start ANC late, usually in their second trimester [29] and hence were enrolled during this period and followed to term on a monthly follow-up basis. Based on their dietary diversity status, they were assigned to either exposed (inadequate dietary diversity) or unexposed (adequate dietary diversity) groups at a ratio of 1:1: and were followed until term.

Sample size was calculated using the Open Epi Kelsey statistical software, considering the following assumptions: a 95% significance level (two-sided), 80% power, and 37% anemia prevalence [28] among exposed and an anticipated 10% lower prevalence of anemia among unexposed pregnant women. This yielded a total of 168 participants per arm, and to allow for a 20% attrition by the end of the study, a sample size of 420 was required. All women who were pregnant and permanent residents of the study area, with no known medical, surgical, or obstetric problems, and who were willing to attend routine Antenatal care (ANC) visits were included to the study.

At enrollment, a 24-h WIDD score was collected from the pregnant women by use of the Food and Agriculture Organization (FAO) guidelines [14], and participants were then divided into “adequate” (WIDD score < 4) or “inadequate” (WIDD score ≥ 4) groups.

Based on FAO guidelines for measuring household and individual dietary diversity [30] as well as synthesizing available research results [26], we used the following nine food groups to calculate the WIDD score: (1) cereals, roots, and tubers [2]; dark-green leafy vegetables (DGLV); (3) vitamin A-rich fruits and vegetables; (4) other fruit and vegetables; (5) legumes and nuts; (6) meat, poultry, and fish; (7) organ meat; (8) dairy; and (9) eggs. A woman was described to consume a certain food group if she took at least a smallest portion equivalent to about 15 g of a food in the last 24 h and consistently (at least in three of the four visits) during follow-up. Dietary diversity scores collected during each visit were calculated by summing the number of food groups consumed by pregnant women over the 24-h recall period.

To look at associations between consumption of specific food groups and anemia, and based on literature [30–32], the nine food groups were further re-categorized into five major food groups for ease of comparison and optimizing adequate cases in each category, as some foods like fish and meat were hardly consumed in the study area leaving few cases for computation. Accordingly, we created the following five food categories re-categorized using iterative approach technique combining similar food groups together: (1) All animal source foods (meat and meat products, dairy, and eggs), (2) meat and meat products as a separate group, (3) all vegetables including vitamin A-rich, (4) all fruits including vitamin A-rich, and (5) legumes and nuts. We created the “meat and meat products” groups separately as meat is a rich source of iron and increases bioavailability through efficient pathway for the intestinal uptake of ferritin, derived from meat-based dietary sources, which involves lysosomal dissolution of the ferritin core to release the iron [33, 34],

Data on the socio-economic characteristics and DD were collected at baseline using a pre-tested questionnaire that was adapted from the Ethiopian Demographic and Health Survey and the FAO [27, 35]. The questionnaires were pre-tested in a similar setting. Data was collected by 24 well-trained and experienced midwives who work permanently in the antenatal care service provision units of health centers in the community.

The pregnant women were weighed during each ANC visit from enrollment to delivery following the standardized procedures recommended by WHO [34]. Pregnant women were weighed to the nearest 100 g on electronic scales with a weighing capacity of 10–140 kg. Their height was measured to the nearest millimeter with a portable device equipped with calibrated and standardized height gauges (SECA 206 body meter). The mid-upper arm circumference (MUAC) of the left arm was measured to the nearest millimeter with a non-stretch measuring tape.

Hemoglobin measurements were taken twice: once at enrollment and once before delivery (term) using a portable HemoCue (AB Leo Diagnostics, Helsinborg, Sweden). The readings were adjusted for altitude [20], and pregnant women with values below 11.0 g/dl were considered to be anemic [20]. Gestational age was estimated by midwives at the health center, by counting from the last menstrual period and fundal palpation during ANC visits.

All data collectors were experienced (≥ 4 years) midwives with at least a diploma in nursing. They also received 5-day training on participants’ enrollment, follow-up, and the use of a HemoCue for hemoglobin measurements. The training was conducted just before the study and was followed by practical tests to ensure the skills were transferred.

In each of the health centers selected, one supervisor (usually the head) was assigned to oversee data collection. In addition, the investigator made a weekly visit to check the completeness and quality of the data collected.

### Statistical analyses

Data was captured using Epi-data statistical software (3.1). Data was double entered and cleaned and then exported to SPPS (version 20.0) for statistical analyses. Continuous variables were checked for normality using the Kolmogorov-Smirnov test. The WIDD scores for each ANC visit (both at mid and term of pregnancy) were calculated by summing the number of food groups consumed by the individual respondent over the 24-h recall period. Consumption of the nine food groups were compared for anemic versus non-anemic women at term using the chi-square test. For each of the newly created five food groups, as well as maternal socio-demographic, reproductive and dietary and nutritional characteristic associations with risk of anemia during pregnancy were computed using binary logistic regression model. Furthermore; collinearity diagnostics were conducted calculating the eigenvalues for variables in Table 2. For eigenvalues above 15, we used *z* scores of the independent variables in the regression model. We also fitted a multivariate binary logistic regression model to identify specific food groups associated with lower risk of anemia at term of pregnancy. A *p* value of 0.05 was used to determine statistical significance of differences.

Using the nine food groups, minimum cutoff values of WIDD score for lower risk of anemia at enrollment (mid-pregnancy) and term were calculated by using a ROC analysis, followed by a validation of their use as a prognostic marker. We carried out the ROC curve analysis to select the optimal cutoff values for WIDD score associated with the lowest hemoglobin level of anemia (11 g/dl) for baseline and end-line measurements. The area under the curve (AUC) summarizes the predictive power of each indicator across all possible cutoff values for food groups. As a rule of thumb, we considered an AUC ≥ 0.65 to indicate some promise for the indicator.

## Results

A total of 432 eligible pregnant women (216 from each group) were identified prospectively and enrolled during their first ANC visit and were followed to term, of whom 374 (86.3%) completed the study with a balanced dropout rate across both groups. The reasons for the dropping out were mainly discontinuation of the ANC visits (*n* = 28), incomplete data (*n* = 12), or not delivering in a health facility (*n* = 18).

Table 1 presents selected socio-demographic and nutritional characteristics of pregnant mothers who completed the study. From the final cohort of pregnant mothers that remained in the study, a larger proportion (39.3%) were in the age group of 20–24 years and 42.5% have completed primary education and 65% of the women attended three or more antenatal care visits.

**Table 1:** Selected baseline socio-demographic and nutritional characteristics of pregnant mothers in rural Arsi, Ethiopia; stratified by anemia status at term (*n* = 374)

Socio-demographic and nutritional characteristics	Pregnant mothers	
	Anemic^a^ (*n*/%)	Non-anemic^b^ (*n*/%)
Maternal age (years)		
< 20	22 (26.5)	61 (73.5)
20–24	27 (27.6)	71 (72.4)
25–29	50 (38.5)	80 (61.5)
≥ 30	22 (34.9)	41 (65.1)
Educational status		
No formal education	40 (33.3)	80 (66.7)
Primary education	38 (40)	57 (60)
Above primary education	43 (27)	116 (73)
ANC visits completed		
One	7 (41.2)	10 (58.8)
Two	39 (34.8)	73 (65.2)
≥ Three	75 (31.1)	166 (68.9)
Hemoglobin (baseline)		
Anemic	81 (75.7)	26 (24.3)
Non-anemic	40 (15)	227 (8.5)

^a^*N*_1_ = 121, ^b^*N*_2_ = 253; *ANC*, antenatal care

**Fig. 1:**
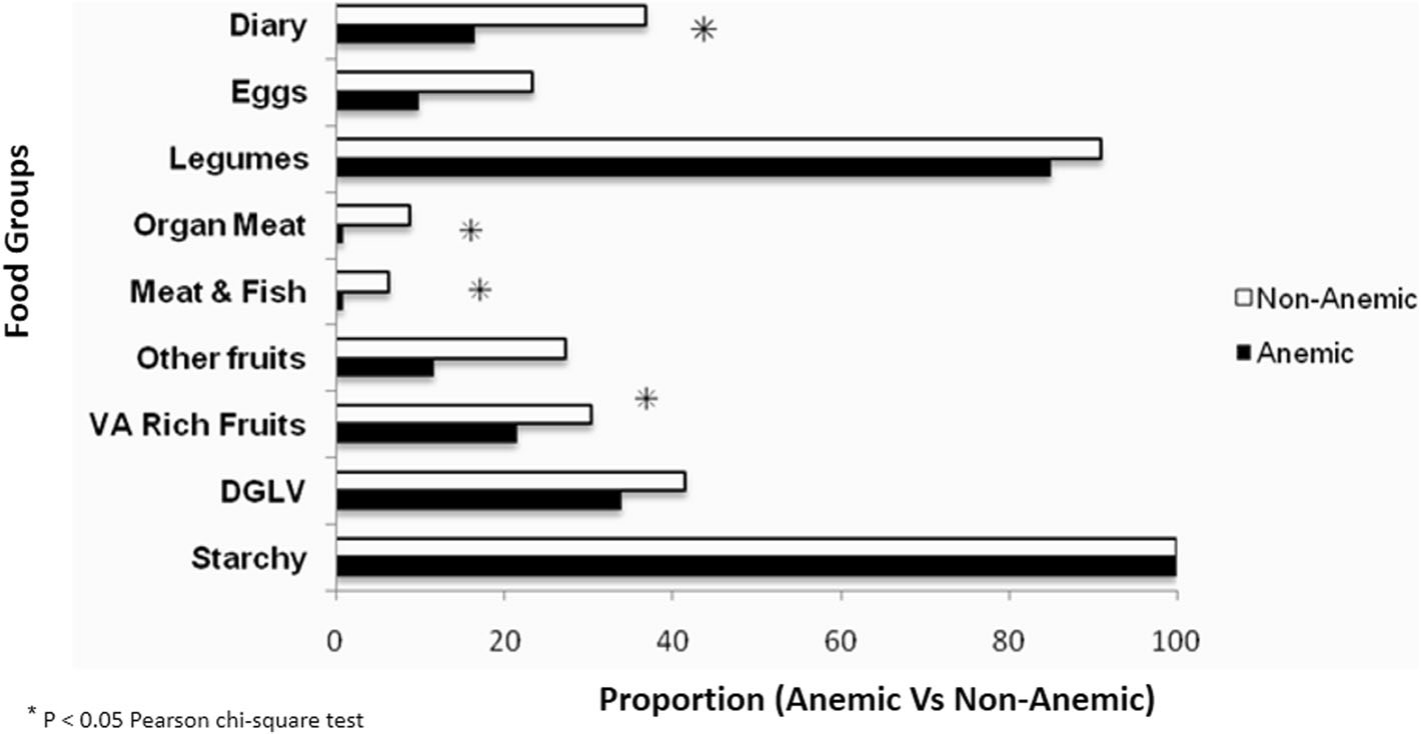
Maternal dietary diversity during pregnancy and anemia status at term in rural Arsi, Central Ethiopia

From the nine food groups consumed during the course of follow-up, consumption of egg, organ meat, meat or fish and diary, and vitamin A-rich plant-based foods was significantly associated with anemia of pregnant women at term (*p* < 0.05) (Fig. 1).

Table 2 presents the findings of a logistic regression model on the association between the newly formed five food group categories with risk of anemia at term. Accordingly, women who did not consume any animal source foods had more than twofold (AOR, 2.36; 95% CI, 1.35, 4.14) higher risk of anemia at term of pregnancy compared with those who did consume animal source foods. Similarly, women who did not consume a diversified diet (consumed three or less food groups) had more than twofold higher risk (AOR, 2.22; 95% CI, 1.09, 4.52) of anemia.

**Table 2:** Logistic regression analysis of pregnant mothers’ key food diversity groups and maternal anemia, rural Ethiopia (*n* = 374)

Food groups (categories) consumed	*n* (%)	Maternal anemia at term	
		COR (95% CI)	AOR (95% CI)
Animal source foods (ASF)^a^			
Yes	141 (37.7)	1	1
No	233 (62.3)	3.25 (1.96, 5.38)*	2.36 (1.35, 4.14)*
Meat and meat products			
Yes	32 (8.6)	1	1
No	242 (91.4)	16.75 (2.26, 124.27)*	7.70 (0.97, 61.03)
Vegetables			
Yes	212 (56.7)	1	1
No	162 (43.3)	1.60 (1.04, 2.48)*	0.74 (0.38, 1.40)
Fruits			
Yes	83 (22.2)	1	1
No	291 (77.8)	2.87 (1.54, 5.34)*	1.97 (0.99, 4.13)
Legumes and nuts			
Yes	333 (89.5)	1	1
No	39 (10.5)	1.91 (0.98, 3.74)	1.32 (0.56, 3.11)
Dietary diversity^b^			
Adequate (WIDD ≥ 4)* Inadequate (WIDD < 4)	186 (49.7) 188 (50.3)	1 3.18 (2.01, 5.03)*	1 2.22 (1.09, 4.52)*

Adjusted for maternal educational status and hemoglobin level

^a^Including all foods of animal origin (meat and meat products, eggs, and dairy)

^b^Out of the original WIDD score classification, based on nine food groups

*This is out of nine food groups

*COR*, crude odds ratio; *AOR*, adjusted odds ratio; *CI*, confidence interval; *ASF*, animal source foods

*Italics* the significance level or *P* <0.05

**Fig. 2:**
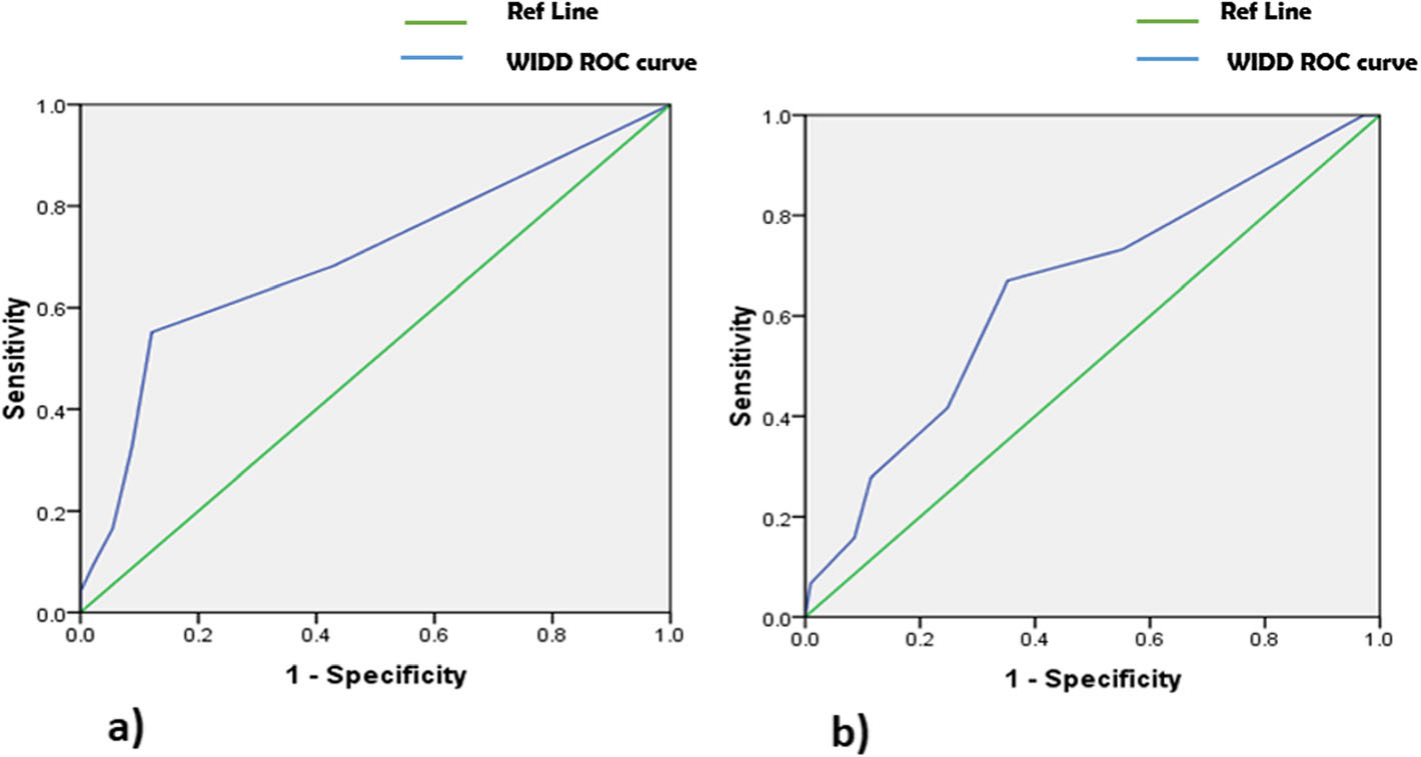
ROC curve of WIDD score relative to maternal hemoglobin level during the second and third trimesters of pregnancy. **a** Optimal cutoff WIDD score value at enrollment (second trimester of pregnancy) was 2.5 (area under curve = 0.699, *p* < 0.05). **b** Optimal cutoff WIDD score value at term (third trimester of pregnancy or term) was 3.5 (area under curve = 0.65, *p* < 0.05)

ROC curves of WIDD score during mid (second trimester) and term (third trimester) pregnancy trimesters were made based on the specificities and the sensitivities of anemia risk to determine the appropriate cutoff value in each pregnancy stage (Fig. 2). The minimum cutoff value for WIDD score associated with lower risk of anemia was 2.5 (three food groups) at mid-pregnancy and 3.5 (four food groups) at term of pregnancy.

Analysis of the association between household and maternal characteristics with anemia risk at term of pregnancy showed that non-possession of radio or mobile phone, maternal educational status, and hemoglobin values at enrollment were associated with risk of anemia at term. Mothers from households without a radio (AOR, 1.93; 95% CI, 1.12–3.39) and/or a phone (AOR 3.14; 95% CI, 1.75–6.62) were more likely to be anemic. Mothers who had completed primary school had a 53% (AOR 0.47; 95% CI, 0.25–0.88) lower risk of anemia compared with those with no formal education. Furthermore, women who were anemic during the second trimester of pregnancy had more than 28-fold increased risk of remain to be anemic (AOR 28.56; 95% CI, 14.33, 56.79) at term compared with non-anemic women (Table 3).

**Table 3:** Logistic regression analysis of pregnant mothers’ selected households and individual nutrition characteristics and anemia risk rural Ethiopia

Maternal characteristics	Number (%)	Maternal anemia at term	
		COR (95% CI)	AOR (95% CI)
1. Land size (hectare)			
No land	133 (35.6)	1	1
< 1	136 (36.4)	0.57 (0.34, 0.96)*	0.60 (0.33, 1.09)
1–2	70 (18.7)	0.66 (0.35, 1.23)	0.77 (0.38, 1.54)
2+	35 (9.4)	0.66 (0.29, 1.46)	1.02 (0.42, 2.47)
2. Presence of radio in the HH			
Yes	270 (72.6)	1	1
No	102 (27.4)	1.48 (0.89, 2.46)	1.93 (1.12, 3.39)*
3. Have mobile/cell phone in the HH			
Yes	286 (76.5)	1	1
No	88 (23.5)	2.55 (1.56, 4.17)*	3.14 (1.75, 5.62)*
4. Have audiovisuals (e.g., TV) in the HH			
Yes	119 (31.8)	1	1
No	255 (68.2)	1.37 (0.85, 2.21)	1.01 (0.57, 1.80)
5. Maternal educational status			
No formal education	120 (32.1)	1	1
Primary	95 (25.4)	0.75 (0.43 , 1.31)	0.47 (0.25, 0.88)*
Primary+	159 (42.5)	1.35 (0.80, 2.26)	0.85 (0.46, 1.57)
6. MUAC (cm)			
< 21 (SAM)	90 (24.1)	1	1
21–23 (MAM)	171 (45.7)	1.86 (1.10, 3.15)*	1.52 (0.75 , 3.11)
23+ (normal)	113 (30.2)	2.66 (1.46, 4.85)*	1.03 (0.43, 2.34)
7. Hemoglobin (g/dl) at enrollment			
< 11 (anemic)	107 (28.6)	1	1
11+ (non-anemic)	267 (71.4)	17.68 (10.14, 30.80)	28.56 (14.33, 56.79)*

*COR*, crude odds ratio; *AOR*, adjusted odds ratio; *CI*, confidence interval; *HH*, household; *MUAC*, mid-upper arm circumference; *SAM*, sever acute malnutrition; *MAM*, moderate acute malnutrition

*Italics* the significance level or *P* <0.05

## Discussion

In this analysis, using the ROC analysis, we aimed to determine minimum cutoff values for WIDD score associated with lower risk of anemia at mid and term of pregnancy, for potential use in a low-income settings. We also fitted a multivariate binary logistic regression model to identify specific food groups associated with lower risk of anemia at term of pregnancy. Accordingly, parallel to the increase in proportion of pregnant women being anemic from mid to term of pregnancy (from 28.6 to 32.4%), the minimum WIDD score cutoff value associated with lower risk of anemia was higher at term of pregnancy compared with mid-pregnancy (four versus three), indicating that pregnant women need a more diverse diet as pregnancy progresses. Not consuming animal source foods, pre-existing anemia and low DD during pregnancy were associated with risk of anemia at term.

To the best of our knowledge, this is the first study reporting new metrics of DD cutoff values predicting risk of anemia during various stages of pregnancy. Given the dynamic nature of pregnancy, we employed a longitudinal prospective study that best suites the condition. The study also reported results of pregnancy and dietary diversity using a rigorous design from a low-income setting where evidence related to the issue is hardly available.

The study also has some limitations that need to be taken into consideration when interpreting the findings. Firstly, we used the FAO WIDD score to measure dietary diversity, which is designed as a proxy measure of dietary quality rather than health outcomes such as anemia. Also, the tool also has nine food groups and our study was not adequately powered to test all these food groups and several thresholds of WIDD score. Secondly, unlike low (2.2% %) rural literacy levels in Ethiopia [29], nearly two out of five (42.5%) women in our study had completed primary school education indicating a relatively better educational status of mothers enrolled in the study that may not be a true reflection of the source population. Thirdly, as mothers participating in this study were drawn from a health facility, selective participation could have occurred thereby leading to biased community level estimates. However, it has been shown that selection bias in cohort studies primarily arises from loss to follow-up rather than to non-response at baseline [36, 37].

Based on WHO classification [38], the magnitude of anemia observed among the pregnant mothers in our study is classified as moderate public health problem. Such levels of anemia, particularly during late periods of pregnancy, are not unexpected in a resource limited setting. Several observational studies in Ethiopia [38–41] and elsewhere [15, 42] have reported even a higher (> 50%) prevalence of anemia during pregnancy, showing the perpetuation of the problem as one of the most important micronutrient deficiencies in the world. Furthermore, in addition to dietary and other health-related risk factors predisposing pregnant women during later periods of pregnancy to elevated risks of anemia, physiologic dilutions will also worsen the problem.

On the other hand, the observed high burden of anemia during pregnancy could be attributed to poor dietary and health care practices as well as other socio-cultural barriers to the intake of micronutrient-rich foods. Evidence shows that the diets of communities in LMIC in general, and that of pregnant women in particular, are often monotonous and predominantly based on plant-based food items with little consumption of micronutrient-dense animal source foods, fruits, and vegetables [43, 44]. Similarly, the level of uptake and compliance to micronutrient supplements containing iron or iron-folic acid (IFA) remain unsatisfactory in many similar settings, including Ethiopia [45–48]. Cultural taboos and dietary practices may also restrict mothers from consuming the available iron-rich foods like meat or food that enhances absorption of iron from food such as fruits and vegetables [27].

The analysis conducted using the ROC curve uncovered a new metrics or critical lower limits (cutoff values) of food groups with a potential for scale for use by frontline agri-nutrition actors in similar settings. As such, a minimum WIDD score of three and four was found to be predicting lower risk of anemia during mid (second) and term (third) trimesters of pregnancy, respectively.

Moreover; the regression analysis using food groups as predictors indicated that non-consumption of ASF and/or diversification of diets during pregnancy were associated with higher risk of anemia during pregnancy. The finding is consistent with similar previous studies [26, 47].

The study also indicated potential association of some household and maternal demographic characteristics with risk of anemia during pregnancy. Not owning a radio and/or a mobile phone was associated with a higher risk of anemia at term. This is consistent with findings of previous studies that showed better health status and health care service utilizations being associated with effective use of information education and communication (IEC/BCC) materials including use of mobile and radio technologies.

In conclusion, the overall prevalence of anemia during pregnancy was moderate in the area, but increased from 28.6 to 32.4% from mid to term of pregnancy. Using the ROC curve analysis, the minimum WIDD score associated with lower risk of anemia was higher at term of pregnancy compared with mid-term. Non-consumption of fruits, vegetables, and animal source foods including meat, organ meat, dairy, and egg was associated with higher risk of anemia.

Additional population-based observational and experimental studies with larger sample size and rigorous experimental design are needed to confirm causality of associations, and validating the metrics before population-level policy level recommendations. Local and international policy makers, planners, and programmers should give emphasis to diet-based approaches though encouraging consumption of animal source foods and dietary diversification throughout pregnancy, particularly during the later weeks of gestation to mitigate maternal anemia and associated prenatal consequences in their planning and programming.

## Data Availability

Please contact author for data requests.

## Abbreviations

ANC: Antenatal care
AOR: Adjusted odds ratio
AUC: Area under the curve
ASF: Animal source foods
CI: Confidence interval
BCC: Behavioral change communication
DD: Dietary diversity
IMMANA: Innovative methods and methods
IDA: Iron-deficiency anemia
FAO: Food and Agriculture Organization
IEC: Information education and communication
IFA: Iron-folic acid
LMIC: Low- and Middle-Income Countries
MUAC: Mid-upper arm circumference
ROC: Receiver operating characteristic
WHO: World Health Organization
WIDD: Women’s Individual Dietary Diversity
